# The Council on Chiropractic Education's New Wellness Standard: A call to action for the chiropractic profession

**DOI:** 10.1186/1746-1340-14-23

**Published:** 2006-10-12

**Authors:** Marion W Evans, Ronald Rupert

**Affiliations:** 1 Parker College of Chiropractic Research Institute 2500 Walnut Hill Lane, Dallas, Texas 75229, USA; 2 Dean of Research, Parker College of Chiropractic Research Institute 2500 Walnut Hill Lane, Dallas, Texas 75229, USA

## Abstract

**Background:**

The chiropractic profession has long considered itself to be a preventive science. Recently the Council on Chiropractic Education (CCE) has defined a set of standards that must be implemented at all US chiropractic colleges as of January of 2007. These are specific to wellness measures and health promoting efforts that should be performed by chiropractors. This will mandate traditional health promotion and prevention methods be taught to students at accredited colleges and to practicing chiropractors.

**Objective:**

To present the idea of performing traditional health promotion and wellness-concepts in chiropractic practice as a call to action for clinicians and generate discussion on the topic.

**Discussion:**

This manuscript discusses relevant topics of health promotion and prevention for chiropractors and other practicing clinicians that should be made priorities with patients in order to enhance both patient health and community and population health.

**Conclusion:**

All practicing chiropractors, as well as other clinicians should take these new standards from the CCE as a call to action to begin helping patients address the removable causes of morbidity, disability and premature mortality where they exist, in addition to treating their painful spinal conditions.

## Background

In January of 2006, the Council on Chiropractic Education (CCE) issued a new standard addressing wellness and health promotion in the chiropractic college curriculum. The standard requires that students demonstrate specific clinical competencies and the changes must be in place in all colleges by the beginning of 2007 [1]. For those not engaged in wellness and health promotion activities, the new CCE standards provide an overdue call to action for the profession. The need for integrating wellness in chiropractic practice is universal and applicable regardless of the position one takes in the debate over whether doctors of chiropractic (DCs) serve as primary care providers or simply spine specialists [2,3]. This paper takes the position that no less should be expected from any health care clinician.

### The CCE Wellness Standards

The CCEs Wellness Standards mandate that the student be taught traditional definitions of wellness and health promotion in addition to strategies for disease prevention. The focus is on both individual and community health considerations. Students must learn current accepted principles of health promotion and in addition must demonstrate those skills in the clinical setting. This process includes assessing the patients' health status, screening for risky lifestyle behaviors, and becoming familiar with multiple health outcome instruments. Following patient assessment, the competencies include educating patients regarding the impact of lifestyle on health, providing appropriate recommendations and counseling, and providing the necessary resources to promote health and wellness. With poor lifestyle choices contributing to the major causes of early death in the United States [4], assessing and assisting the patient modify those risky lifestyle behaviors is one of the key goals of the new standards.

## Discussion

### Health Status of Spine Patients

A review of some of the co-morbidity issues that accompany musculoskeletal conditions like low back pain, will demonstrate why chiropractors need to become aggressively active in addressing patient lifestyle and other health promotion and wellness issues. The impact of spine problems on health status has been examined through co-morbidity analysis. In 2000, Fanuele and colleagues [5] reported an observational study of 17,774 patients from the 25 National Spine Network agencies or academic centers. Their goals were to quantify the impact of spinal problems on physical function and to better understand the effects of co-morbid conditions on physical function. In their study population, 46.6% of spine patients had at least one other non-spinal condition or illness. When smoking was considered a co-morbid condition it was number one with hypertension 2^nd^, obesity 3^rd ^and diabetes 4^th^. Fifty-two percent of patients had a primary diagnosis of lumbosacral symptoms and 82% had experienced three or more months of pain. They concluded that society bears a heavy economic burden from patients with spinal conditions and physicians need to recognize that spine patients have significantly more physical morbidity than the US population in aggregate. Fanuele and colleagues stated, "It is likely that the spinal diagnosis, in itself, is mostly responsible for the significant functional disability, expressed by low physical component scores."

A study published in *Pain *by Von Korff and others [6] concluded that after controlling for demographic variables and for co-morbidities, chronic spinal pain was significantly associated with role disability, other pain conditions, chronic diseases and mental disorders. Their information was derived from the household face-to-face National Co-morbidity Survey Replication which was a nationally representative sample (n = 9,282) of respondents age 18 or older. Almost 20% of the US population was estimated to have chronic spinal pain in the prior 12 months with about 30% reporting lifetime prevalence of chronic spinal pain. This chronic spinal pain was more than three times higher in patients who reported other chronic pain as those without these conditions and it was twice as high in patients with a mental disorder. Chronic physical disease associated with chronic spine pain included stroke, hypertension, asthma, COPD, irritable bowel syndrome, ulcers, HIV/AIDS, epilepsy and vision problems. After adjusting for demographic variables the increased risk of a co-morbid chronic physical disease associated with chronic spine pain was 2.0. Among the 40 million Americans who suffer chronic spine pain, 22 million had a co-morbid physical ailment (87% with chronic spine conditions had at least one co-morbid condition). Therefore, spine patients are in need of health education messages at a rate that may exceed that of non-spine patients.

The association of spinal disease with smoking and obesity is also fairly well established [7,8]. Obesity is associated with more severe pain syndromes among spine patients and they suffer greater impairment in functional status [7]. As previously stated, smoking is often the most frequently found condition associated with spine disease [5,8]. These factors should be important to chiropractors as they primarily see back pain and neck pain patients [9]. The average case mix of DCs tends to include a significant amount of chronic spine patients although there is an indication that DCs utilize certain health promotion measures with them such as; exercise recommendations, ergonomic advice and advice on dietary changes [9]. DCs need to place a greater emphasis on the use of common prevention and health promotion methodologies in their practices. It is our opinion that an emphasis on wellness and health promotion is compatible with either the primary care or the "spine care model" of chiropractic and is congruent with national health initiatives and the chiropractic tradition of holism and self-reported prevention practices [9]. This will be described in more detail but should include cancer prevention dietary recommendations, proper exercise recommendations, appropriate screening procedures that are within scope of practice including, but not limited to cardiovascular disease, hypertension, diabetes, breast, prostate and skin cancer screening.

### Gaps in Health Promotion and Preventive Medicine

To complicate the issue of health promotion in health care delivery, studies of medical providers demonstrate a glaring lack of preventive measures in everyday practice. A minority of patients in primary care receive information on general prevention measures [11]. In a U.S. study of 26,878 patients, 73 % of diabetics reported their physician made a recommendation to increase exercise, but only 31% of non-diabetics indicated they received similar exercise recommendations [12]. With primary prevention defined as keeping the healthy, healthy; secondary prevention defined as risk reduction prior to permanent damage and tertiary prevention simply damage control [13], this represents secondary prevention at best. A minority (31%) of non-diabetics [12] are receiving primary prevention messages regarding appropriate exercise which leaves them at risk for development of the disease.

The medical communities' performance relative to advice on smoking cessation is as poor as their results in providing proper exercise recommendations. In a Centers for Disease Control and Prevention document, Fiore and colleagues noted that only about 40% of smoking patients report their physician discussed smoking cessation [14]. The chiropractic field does not seem to fair too well either. In a recent chiropractic study (n = 808), only about 40% of smoking patients at 9 U.S. teaching clinics reported being advised on their last visit about cessation. Even fewer (18%) said they were given information on cessation [15]. If these results from chiropractic teaching clinics are any indication of practice patterns of field doctors, the profession needs to improve significantly.

Manson and a team of researchers at Harvard suggested that there is an escalating pandemic of obesity and sedentary lifestyle habits with more than 300,000 premature deaths in the US alone [16]. This alarming report calls on all clinicians to help address this issue with increased recommendations for exercise, diet and weight loss. The authors noted that if action was not taken, society will not be prepared to contend with either the adverse financial impact or the resulting health care issues associated with this pandemic. The report states that many physicians did not routinely assess weight and physical activity and did not offer appropriate recommendations to the patient in those areas. Most recently an independent report partially funded by the Robert Wood Johnson Foundation stressed these same findings but to an even greater degree [17].

### Chiropractic's Role in Health Promotion

Rupert reported in 2000 [18] that a substantial part of the DCs practice involves regular patients who choose chiropractic care as part of some health maintenance routine. While it may be argued that this is secondary prevention, we feel this is an excellent place to start health promotion practices with patients. Evans [19] described the use of the maintenance visit to the chiropractor as a place to start advising patients on lifestyle behavioral modifications that could reduce preventable diseases. If recommendations on diet, exercise and smoking cessation alone were to be offered to regular users of chiropractic services, a significant difference could be realized in the health of thousands of patients. As clinicians, there is simply no reason not to address the preventable causes of disease among patients. A reasonable place to start would seem to be with patients who are regular consumers of chiropractic services and who see the DC as a credible source for health information.

## Recommendations for the Profession

### Standardized Assessment

In a recent investigation of chiropractic teaching clinics, Hawk and Evans [15] found that there was no standardized method in place to gather information regarding the smoking status of patients. There is undoubtedly a similar lack of standardization relative to collecting other important lifestyle information related to diet, exercise and other factors. This would suggest that one of the first steps for the profession should be to develop or embrace an existing standardized method of health and lifestyle assessment.

Included in the assessment, DCs must ask about smoking status, previous history of smoking, including all types of tobacco use, packs per day and how many years a patient has used tobacco. They must take height and weight on every patient and this should be reassessed when the patient reenters the practice after not having been seen in several months. Body Mass Index (BMI) can be easily assessed once height and weight is known by applying a simple formula and abdominal fat deposition can be observed or measured via the Waist to Hip Circumference Ratio (WHCR) which may be a powerful indicator of cardiovascular risk, particularly in males [20]. Those patients who are overweight need to be encouraged to get the recommended levels of exercise noted by the CDC [21] and should be encouraged to make dietary modifications. The DC should be prepared to show them how this can be achieved and not simply attempt to speak to them about empowerment strategies. This may necessitate networking with other professionals such as registered dieticians, personal trainers, health educators and the patient's family physician. Directing these activities will likely require additional knowledge of health behavioral theories that help identify those patients most likely to make health-related changes. Some resources are available as Table 1 and other valuable sources of information are cited in the references.

**Table 1:** Health Promotion Resources on the Web.

**Nutritional Information** The National Cancer Institutes 5-A-Day site US Surgeon General's site for Overweight and Obesity Australian joint site for nutrition and exercise

**Exercise Information** US Centers for Disease Control and Prevention Exercise site

**Tobacco Cessation Advising** US Surgeon General's web site Australian site

**Health Theory** Theory at a Glance web site (Free NIH Guide to health behavioral theories) US Centers for Disease Control and Prevention

### CDC Exercise Recommendations

The current CDC recommendations for exercise state that physical activity should be performed at least most days of the week preferably each day; 5 or more days a week if moderate intensity is achieved and 3 or more days a week if vigorous intensity is achieved [21]. The CDC further defines moderate levels as those activity levels producing some increase in breathing or heart rate, perceived exertion from walking briskly, mowing the lawn, dancing swimming or biking on level terrain. Vigorous activity is more intense with a large increase in breathing or heart rate to the degree one cannot carry on a conversation. Examples are aerobic dancing, swimming continuous laps, biking uphill or carrying more than 25 lbs. up a flight of stairs. Physical activity, if that of moderate intensity, should be performed in bouts of at least 10 minutes for a total of at least 30 minutes a day for at least 5 days of the week and vigorous exercise should be performed at least 20–60 minutes per session for 3 or more days a week.

### The National Cancer Institutes' 5-A-Day Program Recommendations

The National Cancer Institute's 5-A-Day program suggests that in order to reduce risks of cancer, American's get at least 5–9 servings of fruits and vegetables per day with a minimum goal of 5 [22]. DCs can provide readily available brochures from 5-A-Day that show serving sizes and ways to get more servings into the diet. The Australian government suggests 2–4 servings of fruits per day and 4–8 servings of vegetables per day [23] which is probably a better goal to stress with patients for optimal returns on this dietary investment. These servings generally total 4.5–5 cups per day and it is stressed that one get a variety of colored fruits and vegetables in the mix. Brochures on what constitutes a serving are available from the 5-A-Day website and also from 5-A-Day coordinators in each state usually free of charge. Any wellness or health promoting health care practice should become familiar with, and utilized these basic recommendations. General information on diet including reduction of foods that are considered health risks and increases in foods that are considered health promoting should be part of routine practice for DCs if we are to address the pandemic of obesity and other diet-related diseases. Increasing fruits and vegetables and decreasing fat consumption in the diet can assist in both obesity-related risk and risks of cancer and cardiovascular disease.

### Smoking Cessation

Smoking is the most preventable cause of death and a major co-morbid habit, when listed as a co-morbidity, associated with chronic spine disease [5,8,14]. A cessation message should be given to every smoking patient at every opportunity that is afforded to the DC. Fiore and others detail a plan for advising the patient using the Surgeon General's 5-A's [14]. These include "*asking*" about smoking status which we have suggested be on every intake paper work in the DC office, "*advising*" smokers to make an attempt at quitting, "*assessing*" their willingness to try quitting, "*assisting*" in this process however possible and "*arranging*" to follow up with the patient. Patients who are not willing to consider cessation should still be given information on benefits of cessation and those who are interested in cessation need to be given appropriate information on where to start. Programs and brochures are available from the CDC, outlined by Fiore and others, The American Cancer Society and The American Lung Association. A partnership with the patient's family physician is also strongly recommended for success as new medications that aid cessation efforts are usually beyond the scope of chiropractic care. The main focus should be that when a patient indicates a willingness to attempt smoking cessation, or to change any other unhealthy behavior, the DC must be ready to provide specific information and resources and not simply stress personal empowerment. Again, an understanding of proper health theoretical framing on who is most likely to attempt a behavior change will be needed to be effective and avoid frustration for both clinician and patient.

### Barriers to Success in Promoting Health and Wellness in Chiropractic

The authors in no way intend to imply that patient behavioral change is an easy task. Additionally, we admit there are barriers to getting DC's to offer even relatively simple health education messages. There are several potential barriers to the CCE Standards making it all the way into private practice. First, DC's have not been traditionally trained in health promotion. Many may see the "maintenance chiropractic" visit as wellness-oriented. However, as has been stated here, primary prevention is the prevention of disease before treatment is needed [13 ]. Those patients who choose regular chiropractic care may be a good place to start delivery of prevention messages as they have trusted the DC for this extended level of care for spine problems [19]. Chiropractic care however, in our opinion, is a form of treatment. The proper training in health behavioral theory, preventive priority areas and at least a working-knowledge of Healthy People 2010 [4] will be needed if DC's are to be effective. While this must start in the colleges, DC's in the field will have to adapt. This will likely require additional training.

Second, DC's cannot simply hope to empower their patients by telling them to change their ways. They will have to build a network of resources from information and websites to refer patients to, all the way to partners within their community like the family physician, personal trainer and dietician. We believe this will be crucial to success.

Additional barriers include certain assumptions that are made here that are common to any health education effort but particularly to DC's in practice. Among them is the assumption that DC's will want to serve in this capacity. Cuing a patient to take action is not hard but it may require additional time and effort by the DC and it can be challenging when the patient has no interest or the DC is inadequately trained. Behavioral frame-works that help identify which patients are ready and willing to make changes will be essential for the DC to understand. Another assumption is that DC's will perform health education tasks appropriately and that their patients will be receptive to it from a DC rather than a primary care medical physician from whom many traditionally rely on for "health" advice. And last, it is assumed that patients will follow-through with advice that is given. While this is not the focus of our argument, we acknowledge that behavior change is difficult. Still, we see it vital that DC's deliver those messages and assist patients in any way possible when it comes to making positive behavior changes.

### The Need for Additional Training

Lack of proper training for the DC is something that cannot be overlooked. While the students in chiropractic colleges will eventually get prevention and health promotion in their curriculum, we assume, field DCs will likely have to learn more about this on their own. This can be done via post-graduate seminars where education on the topic is brought to them or through additional health education and health promotion training at a public university. Numerous articles and websites are available for patients and clinicians and a list of some of these has been provided. DCs are encouraged to take up self-directed study in prevention and traditional health education methodologies and become familiar with initiatives aimed at screening, prevention and health promotion.

## Conclusion

The intent of this paper has been to make note of the new CCE standards as a call to action in the promotion of health and provide some initial strategies for the chiropractic profession. It was not meant to be all inclusive regarding how to implement the CCE recommendations. These comments are to stress that patients of chiropractors are likely to suffer chronic spine problems or at least be at risk for development of them. Therefore, according to current studies on chronic spine patients, patients of DCs are at perhaps a greater risk for chronic but often preventable diseases. These risks can be reduced or removed with lifestyle, behavioral modifications that can be directed by the DC. This is particularly important as most patients are not getting these health promoting messages from other physicians. In addition, all patients who are receptive to a health message to change an unhealthy behavior should be able to receive them from their chiropractor.

Promoting health takes time and may require the DC to change the way they allot patient visit times. However, the role of every health care provider should not only be to reduce pain and suffering where possible but should also include messages on prevention of disease and assistance in reduction of risk factors that cause morbidity and mortality in our societies. The failure of DCs and other providers for that matter, to address the removable causes of disease in patients and communities is no longer professionally acceptable. Society can no longer bare the consequences of our failure to act in this area of promoting health. The newest CCE Standards should be seen as a call to action for all DCs and challenge not only the world's chiropractic teaching institutions, but every practicing chiropractic physician to rise to the occasion. The basic suggestions made here are simple and can take a minimal amount of time to implement but can make a significant contribution to our patients' health. It is absolutely necessary for the profession as a whole to adopt these standards immediately. Anything less would be a disservice to the patients who have placed their health in our trust.

## List of Abbreviations

**BMI**-Body Mass Index

**CCE**-Council on Chiropractic Education

**CDC**-US Centers for Disease Control and Prevention

**COPD**-Chronic Obstructive Pulmonary Disease

**DC(s)**-Doctor(s) of Chiropractic

**HIV/AIDS**-Human Immune-Deficiency Virus/Acquired Immune Deficiency Syndrome

**5A's**-The US Surgeon Generals' anacronym on asking and advising smokers on cessation

**5-A-Day**-The US National Cancer Institutes initiative to get Americans to eat 5 serving of fruits and vegetables per day

**WHCR**-Waist to hip circumference measurement

## Competing interests

The author(s) declare that they have no competing interests.

## Authors' contributions

ME and RR both contributed to the overall content and writing of this manuscript.
